# Haplo-hematopoietic stem cell transplantation and immunoradiotherapy for severe aplastic anemia complicated with nasopharyngeal carcinoma: A case report

**DOI:** 10.1515/biol-2025-1134

**Published:** 2025-07-11

**Authors:** Yanting Gao, Yun Zhang, Hong Wang, Jingjing Xiang, Xiangping Wu, Qinghong Yu

**Affiliations:** Department of Hematology, The First Affiliated Hospital of Zhejiang Chinese Medical University (Zhejiang Provincial Hospital of Chinese Medicine), Hangzhou, Zhejiang, China; Division of Radiology, The First Affiliated Hospital of Zhejiang Chinese Medical University (Zhejiang Provincial Hospital of Chinese Medicine), Hangzhou, China; Department of Pathology, The First Affiliated Hospital of Zhejiang Chinese Medical University (Zhejiang Provincial Hospital of Chinese Medicine), Hangzhou, China

**Keywords:** severe aplastic anemia, nasopharyngeal carcinoma, haploidentical hematopoietic stem cell transplantation, nimotuzumab, radiotherapy

## Abstract

Severe aplastic anemia (SAA) and nasopharyngeal carcinoma (NPC) are two different diseases and are life-threatening if left untreated. The co-occurrence of SAA and NPC is rare and presents a complex therapeutic paradox. This study reports a unique case of a patient diagnosed with both SAA and NPC. The patient initially underwent haploidentical hematopoietic stem cell transplantation (HSCT) to achieve rapid hematologic recovery, followed by nimotuzumab + radiotherapy for carcinoma treatment. This case suggests that sequential haploidentical HSCT and radiotherapy may represent a promising therapeutic strategy for patients with coexisting SAA and NPC.

## 1 Introduction

Acquired aplastic anemia is a rare hematological disorder characterized by hypocellular bone marrow (significantly reduced number of cells) and pancytopenia (deficiency of blood cell components, i.e., white cells, red cells, and platelets) due to autoimmune-mediated destruction of hematopoietic stem cells and progenitors. Aplastic anemia constitutes a potentially fatal bone marrow failure syndrome, often leading to early mortality if untreated. Management strategies for severe aplastic anemia (SAA) include hematopoietic stem cell transplantation (HSCT) and immunosuppressive therapy (IST) as the two principal modalities [1].

Nasopharyngeal carcinoma (NPC) is a distinct and aggressive malignancy of the head and neck region originating from the epithelial lining of the nasopharynx. Therapeutic interventions for NPC typically include brachytherapy, nasopharyngectomy, radiosurgery, intensity-modulated radiotherapy, stereotactic radiotherapy, or combinations of radiotherapy and surgery, sometimes in conjunction with chemotherapy [2].

Concurrent presentation of SAA and NPC poses significant therapeutic challenges due to the inherent risks associated with treatment modalities for each condition. This study reports a uniquely rare case of successful sequential therapy with haploidentical HSCT followed by radiotherapy in a 28-year-old male patient diagnosed with both SAA and NPC at the same time.

## 2 Case report

Thirteen years ago, at 15 years of age, a man presented with pancytopenia was diagnosed with aplastic anemia based on bone marrow aspiration and biopsy. The patient was initially treated with cyclosporine A (CSA) for 3 months, which was discontinued due to lack of response. He required frequent blood transfusions and maintained a stable but severe cytopenic condition over the ensuing 13 years: white blood cell (WBC) counts between 0.8 × 10^9^ and 1.2 × 10^9^/L, absolute neutrophil count (ANC) between 0.3 × 10^9^ and 0.7 × 10^9^/L, hemoglobin between 30 and 40 g/L, and platelet counts between 10 × 10^9^ and 18 × 10^9^/L.

**Figure 1:**
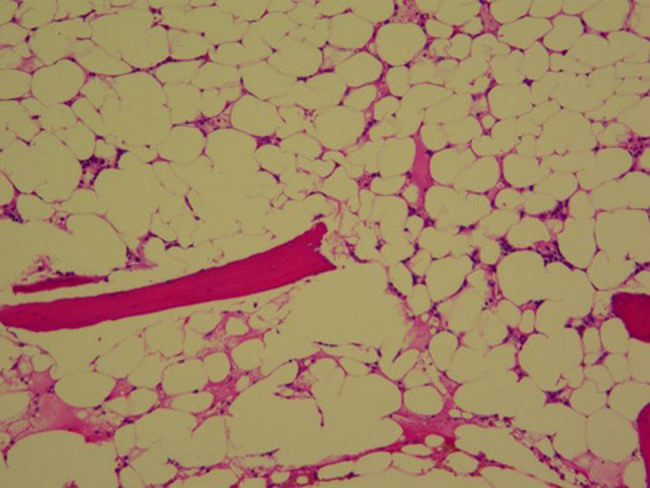
Bone marrow biopsy: marrow cellularity is low to 10% with a few erythroblasts and bone marrow megakaryocytes(hematoxylin and eosin stain, 200×).

In February 2021, he was admitted to the Department of Hematology with progressive pancytopenia, showing WBC 0.7 × 10^9^/L, ANC 0.1 × 10^9^/L, hemoglobin 48 g/L, platelets 5 × 10^9^/L, and absolute reticulocyte count 6.7 × 10^9^/L. Blood Epstein–Barr virus (EBV) and cytomegalovirus nucleic acids were tested negative. Chromosome breakage testing excluded Fanconi anemia, and fluorescence-labeled aerolysin assay by flow cytometry revealed no paroxysmal nocturnal hemoglobinuria clone. Bone marrow karyotype was normal (46, XY), and aspiration and biopsy revealed a hypocellular marrow (<10%) without cytogenetic abnormalities (Figure 1). A diagnosis of SAA was confirmed.

**Figure 2:**
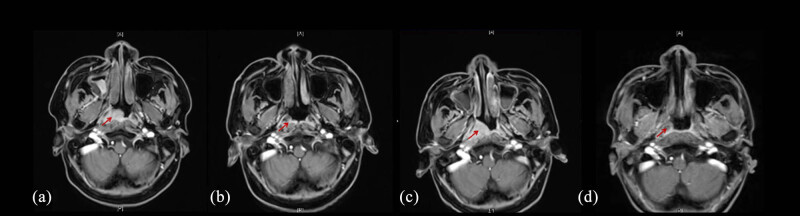
MRI scans before and after treatment. (a) The arrow shows a soft tissue mass at the right pharyngeal recess (2021.5.3). (b) The arrow shows that the mass significantly shank at 1 month posttranspantation (2021.7.8). (c) The arrow shows that the mass enlarged again at 1.5 months posttranspantation. (d) The arrow shows that the mass was disappeared at 11.5 months post-transplantation (6 months after immunoradiotherapy).

**Figure 3:**
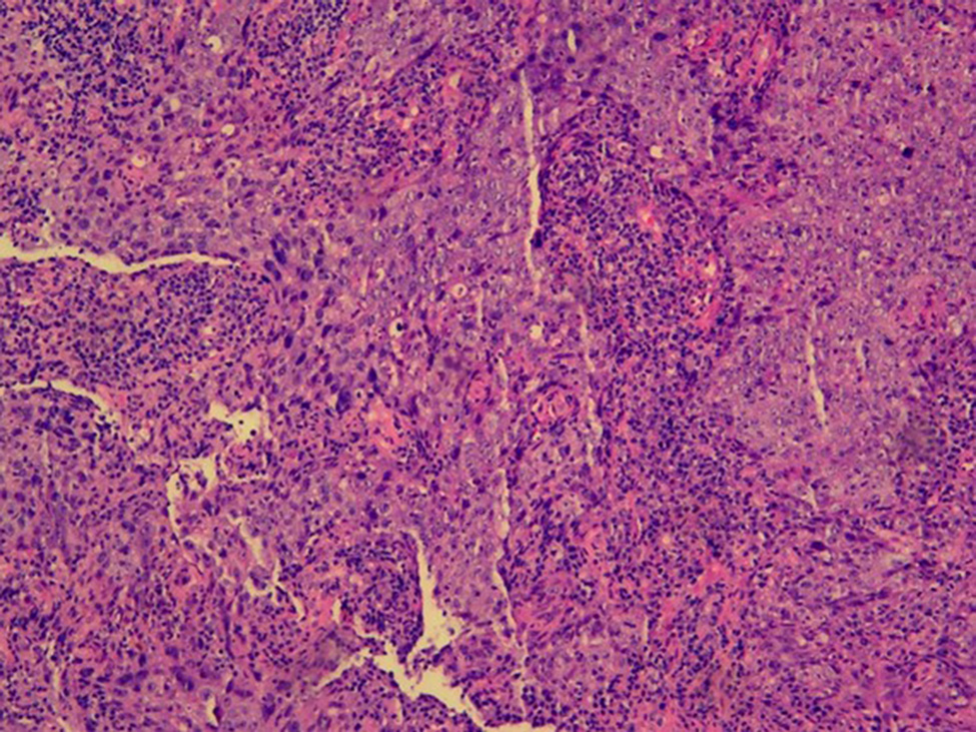
Right nasal cavity mass biopsy. Histology slide confirming NPC (hematoxylin and eosin stain, 200×).

The patient was restarted on CSA combined with testosterone undecanoate. After 2 months, red cell and platelet transfusion dependence persisted, and the patient developed intractable epistaxis (nose bleeding). Nasal sinus magnetic resonance imaging (MRI) revealed a soft tissue mass (Figure 2a). The endoscopic biopsy of the lesion confirmed a diagnosis of NPC (Figure 3). He has no smoking history and no family history of NPC [3]. He has no history of hepatitis B. As severe pancytopenia is a contraindication for tumor therapy, a rescue strategy was designed: initial hematopoietic reconstitution through allogeneic hematopoietic stem cell transplantation (allo-HSCT), followed by definitive cancer therapy.

A haploidentical donor was arranged for the transplant, but the patient exhibited donor-specific anti-HLA antibody (DSA) positivity (anti-DQB1*06:09, mean fluorescence intensity [MFI] = 11518.4). A desensitization protocol combining plasmapheresis, rituximab, and bortezomib was initiated. Preconditioning consisted of fludarabine 30 mg/m^2^/day for 5 days, cyclophosphamide 50 mg/kg/day, and rabbit anti-thymocyte globulin 2.5 mg/kg/day for 4 days. For graft-versus-host disease (GVHD) prophylaxis, CSA (target level 150–250 ng/mL), mycophenolate mofetil (MMF; 0.5 g twice daily), and methotrexate (15 mg/m^2^ on day +1 and 10 mg/m^2^ on days +3, +6, and +11) were administered. Stem cells were harvested from bone marrow and peripheral blood.

Neutrophil engraftment (>0.5 × 10^9^/L) was achieved by day +15, and platelet engraftment (>20 × 10^9^/L without transfusion) by day +24. Short tandem repeat testing at day +30 confirmed 97.4% donor chimerism. Remarkably, complete remission of intractable epistaxis was achieved post-transplantation, and slight tumor regression in the nose was observed by MRI (Figure 2b). Two months later, peripheral blood counts improved: WBC 3.6 × 10^9^/L, ANC 2 × 10^9^/L, hemoglobin 80 g/L, and platelets 60 × 10^9^/L. Neither GVHD nor infections were observed. However, the nasal mass showed slight enlargement on MRI (Figure 2c).

Staging according to the International Union Against Cancer/American Joint Committee on Cancer (UICC/AJCC) Tumor-Node-Metastasis system revealed stage II disease (T2N1M0). The patient then underwent radical radiotherapy: IMRT using 6-MV X-rays was delivered to the gross tumor volume of NPC (GTVnx; 70.4 Gy/32 fractions), gross cervical lymph node metastasis (GTVnd; 66 Gy/32 fractions), and primary clinical target volume (PTV-CTV1; 60 Gy/32 fractions). Nimotuzumab (200 mg/week on weeks 1–3) were successfully administered concurrent with IMRT, based on the immunohistochemistry results that the EGFR expression was positive.

**Figure 4:**
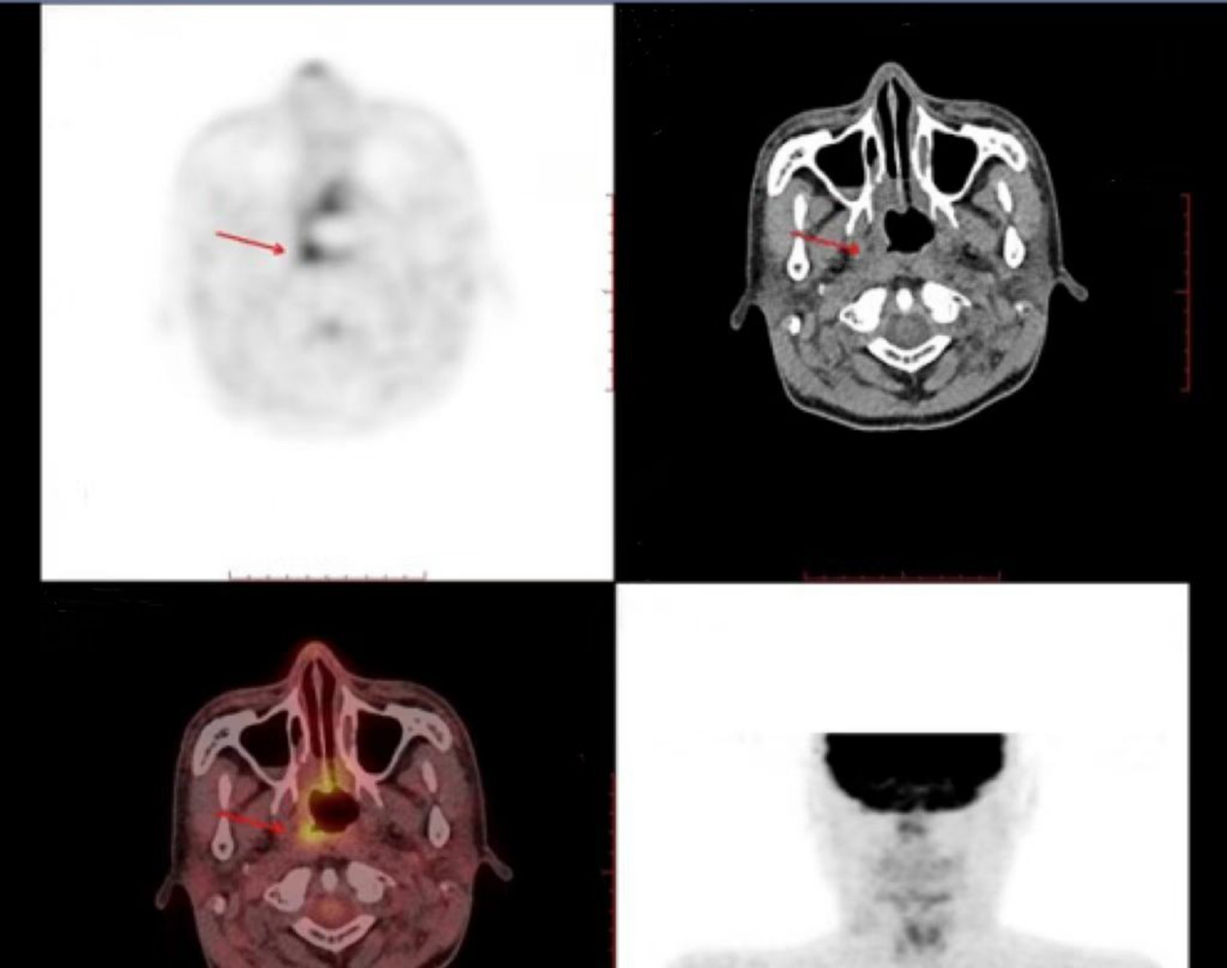
Positron emission tomography-computed tomography scans. The arrow shows that the mass significantly shrank.

**Figure 5:**
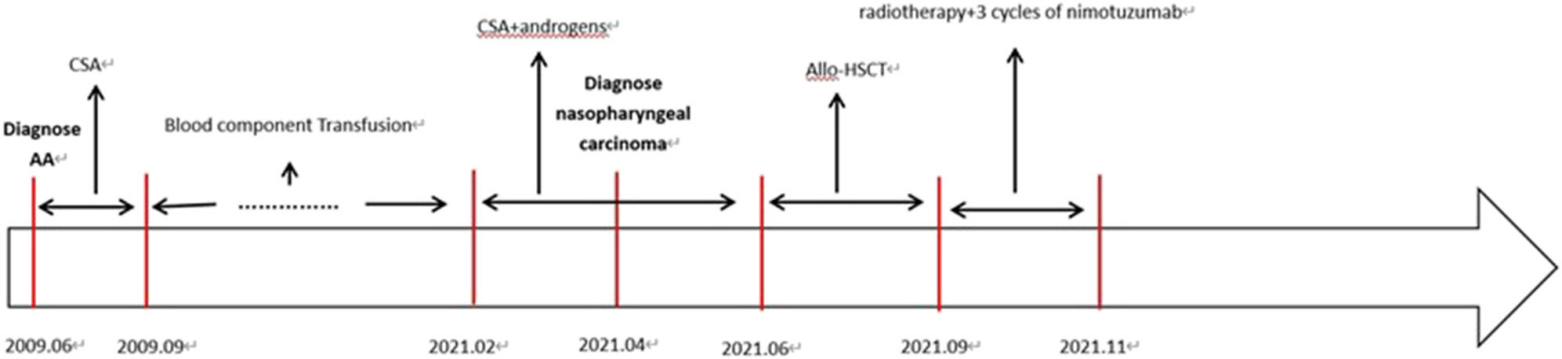
Timeline of treatment.


Transient interruptions of radiotherapy occurred due to treatment-related thrombocytopenia and leukopenia, which were managed with platelet transfusions, thrombopoietin, and granulocyte colony-stimulating factor support. Post-transplantation complications included hypertension and posterior reversible encephalopathy syndrome, secondary to CSA therapy, which was resolved by switching to etanercept and MMF. Eight months post-transplantation, the graft continued to produce red blood cells successfully. The patient remained healthy without GVHD or severe infections. Complete remission of NPC was still maintained at 6 months after immunoradiotherapy and 2.5 years post-transplantation (Figures 2d, 4 and 5).

**Informed consent:** Informed consent has been obtained from all individuals included in this study.
**Ethical approval:** The research related to human use has been complied with all the relevant national regulations and institutional policies and in accordance with the tenets of the Helsinki Declaration and has been approved by the First affiliated Hospital of Zhejiang Chinese Medical University (No. 2024-KLS-457-01).

## 3 Discussion

This case highlights the successful management strategy for acquired bone marrow failure syndrome and carcinoma, involving sequential radiotherapy following HSCT.

The diagnosis of SAA in the patient is clear. In previous treatment experience, ATG + CsA or allo-HSCT is preferred for this type of patient. The patient also has NPC, thrombocytopenia, and recurrent nasal bleeding. The median onset time after ATG treatment is at least 4–6 months, which may delay the treatment of NPC.

Idiopathic acquired aplastic anemia is a sporadic, life-threatening disease that is predominantly immune-mediated. IST is the first-line treatment for SAA patients without an HLA-matched sibling donor or those older than 40 years [4]. The standard IST (ATG + CSA) leads to hematologic responses in about two-thirds of patients and provides overall survival rates >80–85% [5,6,7]. Thrombopoietin receptor agonists (Tpo-RAs) are considered salvage options for SAA patients unresponsive to IST. In a Phase I/II study, eltrombopag monotherapy achieved an overall hematologic response rate of 40% (17 of 43 patients) in relapsed or refractory SAA [8]. Additional clinical data confirmed that eltrombopag is effective as salvage therapy, with overall response rates ranging from 40 to 50% [9]. Based on these outcomes, a three-drug regimen of antithymocyte globulin (ATG), CSA, and eltrombopag has been employed as frontline therapy. In a prospective study evaluating horse-ATG/CSA combined with eltrombopag, 92 consecutive, initially untreated SAA patients were enrolled and divided into three cohorts: cohort 1 received eltrombopag from day 14 to 6 months; cohort 2 from day 14 to 3 months; and cohort 3 from day 1 to 6 months. Eltrombopag dosing was adjusted by age: 150 mg for patients ≥12 years, 75 mg for patients aged 6–11 years, and 2.5 mg/kg for patients aged 2–5 years. Participants of East or Southeast Asian ethnicity received half the standard dose. The complete and overall response rates surpassed those of previous cohorts, with a 58% complete response rate at 6 months and a 97% 2-year survival rate in cohort 3 [10].

The patient, in this case, was 28 years old and lacked an HLA-matched sibling donor; his elder brother was HLA-haploidentical. Although the combination of ATG/CSA and eltrombopag was appropriate, the patient accepted CSA and testosterone undecanoate therapy, declining ATG and eltrombopag due to economic limitations. CSA was initiated at 4 mg/kg/day, adjusted to a target trough level of 150–250 ng/mL, and testosterone undecanoate was administered at 80 mg twice daily. Unfortunately, NPC was diagnosed 2 months after initiation of SAA therapy.

NPC arises from the epithelial lining of the nasopharynx. In 2018, over 129,000 new NPC cases were reported worldwide, with more than 70% occurring in East and Southeast Asia [11,12]. The global age-standardized incidence rate was 3.0 per 100,000 in China compared to 0.4 per 100,000 in white populations [10]. In 2015, the male-to-female incidence ratio in China was approximately 2.5 [13]. Non-keratinizing NPC, which is closely associated with EBV infection, accounts for more than 95% of cases in China [10]. In this case, the tumor was a non-keratinizing subtype and positive for EBV-encoded small RNAs. The overall 5-year survival rate in the 15–45-year age group is approximately 72% [14]. Given the stage II (T2N1M0) classification, the disease was managed as a locoregional malignancy. Radiotherapy remains the primary curative-intent modality for non-disseminated NPC; however, severe cytopenia prohibited immediate initiation. Following multidisciplinary team consultation, the treatment strategy prioritized rapid hematopoietic recovery through HSCT, followed by definitive carcinoma therapy.

SAA can be treated with either allo-HSCT or IST. Allo-HSCT is considered the preferred treatment for SAA. Although long-term overall survival rates are comparable between patients undergoing IST and allo-HSCT, event-free survival is significantly better among those receiving allo-HSCT [15,16]. Importantly, allo-HSCT offers the advantage of more rapid blood cell count recovery compared to IST. Patients younger than 40 years with SAA and a fully HLA-matched sibling donor are recommended to undergo allo-HSCT as first-line therapy [17]. Fully matched unrelated donor (MUD) HSCT has also been adopted as frontline therapy for patients younger than 20 years [18]. Haploidentical HSCT using novel conditioning regimens overcomes disadvantages, including higher risks of graft rejection, severe GVHD, and reduced survival outcomes. Huang et al. conducted a registry-based multicenter study comparing upfront haploidentical HSCT (*n* = 89) and matched sibling donor HSCT (*n* = 69) in SAA. The study reported similar 3-year overall survival (86.1 vs 91.3%, *P* = 0.358) and failure-free survival (85.0 vs 89.8%, *P* = 0.413) between the two groups [19]. In China, the probability of finding an appropriate unrelated donor remains low (∼11%), and donor searches typically require 3–6 months [20]. Approximately 40 years ago, Hattori reported that one of two NPC patients remained healthy for 2 years following autologous bone marrow transplantation in unmaintained remission [21]. Considering these factors, haploidentical HSCT was the most rational and feasible choice for this patient.

A major concern before transplantation was the presence of DSA in the patient’s serum [22,23]. Chang et al. reported that among 345 HSCT recipients, 87 (25.2%) were positive for anti-HLA antibodies, including 39 (11.3%) with DSA. The results showed that the cumulative incidence of primary graft failure (PGF) was 6.4 ± 1.3%. DSA with MFI ≥10,000 was strongly associated with PGF (*P* < 0.001), and DSA MFI ≥2,000 was linked to a significantly higher incidence of graft failure (*P* = 0.005) [22]. The patient’s DSA MFI exceeded 10,000, indicating a significant risk of graft rejection. Although selecting an alternative donor would have been preferable, none was available. Therefore, desensitization was performed concurrently with conditioning therapy according to the MD Anderson Cancer Center protocol [24]. Successful engraftment for leukocytes and platelets was achieved within 1-month post-transplantation. Intractable epistaxis is a common clinical symptom in NPC. In the treatment of NPC, different treatment plans are formulated according to the different stages of development of NPC and the patient’s own tolerance. Clinically, the principles of early- and long-term, positive and rational, comprehensive treatment and individualized treatment measures are mainly followed, and they are treated with radiotherapy, chemotherapy, surgical treatment, etc. Tumor immunotherapy may represent a promising therapeutic approach in NPC [11]. Complete remission of epistaxis was observed immediately after conditioning. Furthermore, slight tumor shrinkage was observed on MRI within 2 months following HSCT. However, carcinoma progression was observed 3 months post-transplantation, suggesting a weak graft-versus-tumor effect and/or suppression by post-transplant IST.

Overexpression of epidermal growth factor receptor (EGFR) is an independent adverse prognostic factor in NPC [25]. Nimotuzumab, a humanized monoclonal antibody targeting EGFR, has demonstrated good tolerability and may improve radiocurability in unresectable advanced head and neck carcinomas [26]. Several small prospective and multiple retrospective trials have supported its clinical benefit [27]. For example, a retrospective study of 257 patients with locoregionally advanced NPC treated with nimotuzumab (h-R3) plus IMRT, with or without chemotherapy, reported estimated 5-year local recurrence-free survival and overall survival rates of 94.3 and 86.2%, respectively [28]. Another retrospective study suggested that nimotuzumab combined with radiotherapy could be a treatment option for stage II NPC patients older than 60 years and intolerant to cisplatin [29]. Although IMRT (II, A) or IMRT combined with chemotherapy (II, B) is the standard recommendation for stage II NPC according to the ESMO-EURACAN Clinical Practice Guidelines [2], this patient represented a special case characterized by poor tolerance to radiochemotherapy and a high recurrence risk due to transplantation and prolonged immunosuppression for GVHD control. Therefore, a synchronous treatment regimen with nimotuzumab and IMRT was successfully administered.

## 4 Conclusion

In 2024, evidence-based guidelines for SAA allo-HSCT were proposed [30]; SAA coexisting with NPC can be successfully managed through sequential HSCT and radiotherapy. Haploidentical HSCT may serve as a feasible and effective alternative when matched sibling donors and MUD are unavailable, even when DSA is positive.
